# EFL university students’ perceptions about cross-cultural presentations *via* videoconferencing

**DOI:** 10.3389/fpsyg.2023.1101334

**Published:** 2023-02-06

**Authors:** Li-Tang Yu, Hsiao-Ping Wu

**Affiliations:** ^1^Department of English Instruction, National Tsing Hua University, Hsinchu, Taiwan; ^2^Department of Educator and Leadership Preparation, Texas A&M University-San Antonio, San Antonio, TX, United States

**Keywords:** cross-culture, videoconferencing, presentation, authenticity, EFL, university students

## Abstract

This study aims to examine EFL university students’ perceptions about cross-cultural videoconferencing presentations for professors in the United States. Nineteen Taiwanese English-as-foreign-language university students who studied in a night program for a bachelor’s degree attended the study. Each of them was free to choose a topic related to Taiwanese cultures and individually made a 25-min presentation plus a five-minute question-and-answer session. After the presentation, they watched recordings of their presentations, completed a five-point Likert-scale attitude survey, and wrote down their reflections. The results showed that the participants were positive about the activity. Based on the analysis of their reflections, the participants acknowledged various aspects of the cross-cultural presentations, such as the opportunities to use English meaningfully, motivation to prepare for the project, and the authentic nature of the interaction. However, the participants had concerns about their language abilities, anxiety, and limited preparation time. Finally, they mentioned the impact the presentation had on them. Pedagogical implications and suggestions for future research are provided.

## 1. Introduction

Language classrooms are a major venue for English-as-a-foreign-language (EFL) learners to get exposure to English (Al Zoubi, 2018). It is important to maximize language-use opportunities within the classroom for EFL learners to practice the target language effectively and efficiently. One of the most common activities in language classes is making presentations (Sirisrimangkorn, 2021).

Language learners can benefit from making presentations. For example, through presentations, learners are able to integrate a variety of language skills, sharpen speaking abilities, prepare themselves for future job applications, develop learner-centeredness, and foster their digital literacy (Al-Issa and Al-Qubtan, 2010). However, EFL learners typically make presentations to their classmates and teachers in class, confining themselves to cohorts within the classroom (Lee and Liu, 2022). Thus, learners simply receive a narrow scope of purpose and audience.

Research has shown that foreign-language learners can achieve better learning performance when they use the language in a meaningful, authentic way (Chamot, 2009; Long, 2015). It is critical to accommodate effective tasks in class to engage students in real-world, authentic communication with the features of purposefulness, reciprocity, synchronicity, and unpredictability (Beatty, 2015). With the advancement of information technology, language learners can obtain more target-language exposure-and-use opportunities than before. Specifically, they can own access to target-language speakers or more competent target-language users by means of computer-mediated communication (CMC) tools outside their regular class. This enhances learner awareness of the audience and fosters second/foreign language proficiency (Hung and Huang, 2015).

Among various CMC applications, videoconferencing provides a platform that closely resembles face-to-face communication without temporal and spatial limitations (Mitchell, 2021). It also enables learners to reach audiences beyond their classmates and teachers and expand their learning environment. Interactive, communicative tasks can be conducted *via* videoconferencing for language learners to use a wide variety of communicative strategies and sharpen their language abilities (Cirit-Işıklıgil et al., 2023), whose effectiveness has been demonstrated in recent review studies (Ibrahim and Hashim, 2021; Yu, 2022).

Acknowledging the importance of authenticity in language-learning activities and the useful affordances of videoconferencing-based CMC on language learning, educational researchers and practitioners have developed numerous inter-cultural communicative videoconferencing activities. The majority of activities was undertaken by students with different first-language backgrounds (Chen and Yang, 2014; Lenkaitis et al., 2019). However, there was little research involving presentations as main tasks in videoconferencing-based interaction. Thus, the researchers developed a cross-cultural presentation project for EFL university students in Taiwan and invited university teachers in the United States as the audience. The students presented to the teachers in the United States an impressive characteristic of Taiwanese culture and respond to the teachers’ questions at the end of their presentation. The current study reports the impact of the cross-cultural videoconferencing project on Taiwanese students’ English-presentation learning.

### 1.1. Cross-cultural videoconferencing projects for language learning

Cross-Cultural videoconferencing projects have been implemented in the field of second-language teaching. The use of videoconferencing has been supported by second-language learning theories. Videoconferencing, by nature, creates a multimodal environment with audio, visual, and textual messages, which is similar to face-to-face communication. Spontaneity and interaction are created naturally. Language learners are able to undertake more negotiations of meaning and boost their second language development (Gass and Selinker, 1994; Chun and Plass, 2000; Long, 2015).

Moreover, supported by sociocultural theories, videoconferencing facilitates its users to build a learning community. With the impact of videos, users gain confidence and reduce the sense of isolation (Hampel and Hauck, 2004). Language learners can reach native speakers or more competent target-language users through videoconferencing and construct knowledge through dialogues (Vygotsky, 1978). Consequently, communicative competence can be developed more effectively (Saito and Akiyama, 2018; Tecedor and Campos-Dintrans, 2019).

Research has reported a variety of cross-cultural videoconferencing projects involving second-language learners. Freiermuth and Huang (2020) have further advocated that telecollaborative videoconferencing activities are beneficial to the enhancement of learner inter-and intra-cultural understanding. Learners also gain more exposure to cultural content and develop their inter-cultural competence (Tecedor and Vasseur, 2020). They could become sensitive to the target culture of their videoconferencing partners when they participate in intercultural interaction (O’Dowd, 2000).

Positive learning feedback on cross-cultural videoconferencing tasks has been reported. Jauregi and Bañados (2008) conducted a cross-continent videoconferencing project and examined the feedback from the participating learners in the Netherlands and Chile. They found that both sides displayed increased intercultural understanding at the end of the project. Similarly, in a recent study, Hsu and Beasley (2019) found that Taiwanese university students had highly positive attitudes toward intercultural videoconferencing tasks, and their intercultural competence was bolstered. Lenkaitis et al. (2019) analyzed videoconferencing recordings of Spanish and English students’ interactions and concluded that videoconferencing telecollaboration was useful in enhancing collaboration and inter-cultural development. Videoconferencing telecollaboration provides learners with an opportunity to explore language-culture intersections in L2 learning and teaching. Finally, in Vurdien and Puranen’s study (2020), a connection between students in Spain and Finland was built *via* videoconferencing. They used English to discuss different aspects of their cultures. Positive student attitudes toward inter-cultural interaction were reported.

Cross-cultural videoconferencing creates natural, authentic interaction occasions for target-language use. For example, university EFL students in Ecuador became more confident, motivated, and fluent in speaking English after finishing an international language-exchange program (Sevy-Biloon and Chroman, 2019). Furthermore, university EFL students in Saudi Arabia had higher speaking proficiency after a 12-week intercultural videoconferencing project in comparison with those who did not participate in the project (Alshahrani, 2016). University Spanish learners in the United States who paired with target-language dominant speakers in a 12-week videoconferencing project perceived more engagement in communicative tasks and exposure to varied language forms and feedback, thus benefiting second-language development (Hetrovicz, 2021, 2022). Cross-cultural videoconferencing grants the target-language use in a meaningful way, which fulfills foreign-language learners’ needs.

As the previous literature suggests, second-language learners can boast a plethora of second-language skills and abilities in cross-cultural videoconferencing projects. However, to the researchers’ knowledge, little research has yet examined second-language learners’ perceptions of presentation, which is an important academic activity (Prevoo et al., 2016), as videoconferencing tasks and how this may affect their second-language learning experience in the context of videoconferencing. Thus, two questions are raised in the current study:

- What are the EFL university students’ attitudes towards the cross-cultural videoconferencing presentation project?
- What are the EFL university students’ experiences in the cross-cultural videoconferencing presentation project?

## 2. Methodology

### 2.1. Context and participants

This study was conducted in a continuing-education program of a comprehensive, private university in northern Taiwan. The program is designed for students who have full-time jobs while pursuing a bachelor’s degree. The university has 11 academic departments and 10 bachelor-degree programs with approximately 4,000 students. Its courses start from the late afternoon to the night to meet students’ schedules. Students are awarded a bachelor’s degree after they obtain mandatory course credits.

Nineteen junior students (14 females and 5 males) in the Department of English participated in the study. They had learned English for at least 11 years with B1 Common European Framework of Reference for Languages (CEFR) proficiency level. They were motivated to sharpen their English abilities to satisfy job demands in their daytime workplaces. They enrolled in a mandatory two-credit English Conversation course with weekly two-hour classes in the 18-week semester. The course was designed to strengthen students’ oral proficiency and support them in expressing themselves in English. Their English proficiency levels were at pre-intermediate levels.

All students were informed of the research purpose and rights. Their identified information was removed to ensure anonymity and confidentiality. All of them provided consent to participate in the research.

### 2.2. Cross-cultural presentation project

The Cross-cultural Presentation Project was implemented in the course in the second half of the semester during 2020–2021. A connection between the university in Taiwan and that in the United States was established and coordinated by the researchers of the current study. The participants took turns making a 30-min presentation (including a five-minute Question & Answer session) whose topic was related to their culture, aiming to prepare themselves to express their local cultures in English (Yeh, 2011). Authentic audiences of the presentations came from the faculty members who worked in the bilingual program in Southern Texas in the United States. They are either native, near-native, or bilingual speakers who can produce the language spontaneously. They evaluated the participants’ presentation performance and posed questions or gave feedback to the participants in the Question & Answer session.

There were three phases in the project throughout the 18-week semester. To begin with, the first stage (Preparation) took 10 weeks to give presentation skills to the participants. At the end of the Preparation phase, both the researchers and the faculty in the United States communicated with each other and discussed how the presentation activity would be undertaken. Then, both discussed a presentation rubric and discussed what needed to be revised in the rubric. In the second phase (Presentation) for the following 6 weeks, two or three participants were assigned to present their chosen topic each week. The participants’ presentations were video-recorded. The faculty also evaluated the participants’ performance during their presentation and sent evaluation scores directly to the researchers. The score accounted for 30% of the course grade. Finally, in the final 2 weeks of the semester, which was the third phase (Reflection), the participants received recordings of their presentations and wrote down their reflections on the project.

### 2.3. Instruments

Two instruments were adopted to explore student perspectives on the cross-cultural videoconferencing activity. Firstly, a survey about their attitudes towards the cross-cultural presentation task was designed. There were eight five-point Likert scale question items (1, strongly disagree, 2: disagree, 3: neutral, 4: agree, and 5: strongly agree) regarding the perception of the presentation task, understanding of their learning, culture, as well as motivation, and willingness to conduct the task. The Cronbach’s alpha value was 0.89, which was good (Taber, 2018). Secondly, a reflection essay with prompt questions was designed. Prompt questions were all open-ended, guiding participants to reflect on the process of the activity, including their preparation, reflection, and feedback about the activity.

### 2.4. Data collection and analysis

At the end of the Preparation stage, both the participants and the faculty in the United States received the presentation rubric and understood its content. During the Presentation phase, the participants’ presentations were screen-recorded. The faculty also evaluated participant performance during the presentations and directly sent evaluation scores to the researchers. Then, in the Reflection phase, the participants received their video-taped recordings. They watched their own recordings, self-evaluated their performances by using the rubric, filled out the attitude survey, and completed their reflection essay.

The data were from two sources: the attitude survey and the reflection essay. Both data were collected for triangulation, thus analyzing multiple sources of evidence to confirm the accuracy of information (Friedman, 2012). Descriptive statistics was used to investigate the response to the attitude survey items. Then, a qualitative analysis was conducted to examine students’ reflection essays through content analysis. The researchers read the reflection essays carefully and used QDA Miner Lite for analysis. A constant comparative analysis (Corbin and Strauss, 2015) was conducted on data to find themes emerged from the students’ reflections on their videoconferencing presentation experience. Meaningful categories emerged from the essay responses were identified to fit the theme. Three major themes with a total of eight categories were thus developed. To dissolve any discrepancies about the coding outcome, regular discussions between the researchers of the study were held. Finally, peer debriefing (Lincoln and Guba, 1985) was conducted with a professor with a specialty in technology-enhanced language learning to verify the trustworthiness of data interpretation.

## 3. Results

### 3.1. EFL university students’ attitudes towards the cross-cultural presentation

To explores EFL university student attitudes towards the cross-cultural videoconferencing presentation project, the survey was conducted at the end of the project for an overall understanding of the participant attitudes towards the intercultural videoconferencing presentation. Details of the attitude survey are presented in Table 1. The results showed that at least 13 of 19 participants (more than 68%) agreed with all the survey items. Thus, most of the item means were above four out of a five-point Likert scale, displaying that the students were positive about the activity. Only two items (“I like the presentation activity” and “I would like to join this presentation activity again”) had the lower means, but still close to four. The top two items that the participants agreed on the most were their recognition of the effect the project had on their oral-English proficiency and the development of their own intra-cultural understanding. Particularly, the majority of the participants (more than 90%) considered the presentation activity helpful for their speaking proficiency development, and most of the participants (around 89.5%) viewed the presentation activity as encouraging in fostering their oral English proficiency.

**Table 1:** Attitude towards the intercultural videoconferencing presentation activity.

Item	1	2	3	4	5	M	SD
The presentation activity helped my oral English proficiency development	0	0	5.3%	52.6%	42.1%	4.37	0.60
The presentation activity encourages me to develop my oral English proficiency	0	0	10.5%	47.4%	42.1%	4.32	0.67
From the presentation preparation, I better understand my own culture	0	0	15.8%	36.8%	47.4%	4.32	0.75
I thought that the presentation activity made English speaking meaningful	0	0	15.8%	36.8%	47.4%	4.32	0.75
I learned something from my classmates’ presentations	0	0	21.1%	31.6%	47.4%	4.26	0.81
The interaction with the teachers in the United States was helpful in developing my oral English proficiency	0	0	21.1%	42.1%	36.8%	4.16	0.76
I like the presentation activity	0	5.3%	31.6%	26.3%	36.8%	3.95	0.97
I would like to join this presentation activity again	0	5.3%	26.3%	36.8%	31.6%	3.95	0.91

*N* = 19; 1: strongly disagree, 2: disagree, 3: neutral, 4: agree, and 5: strongly agree; the percentages were rounded up to the third decimal point.

The bottom two items with less student support were concerned with their enjoyment and willingness to participate in such a presentation. Twelve of the participants (around 63%) liked the presentation activity, and 13 of them (around 68.4%) wanted to join the presentation activity again. This suggests that they generally appreciated the learning opportunity represented by the activity, despite having some concerns.

### 3.2. EFL university students’ perspectives on the cross-cultural presentation

Despite having positive attitudes toward the presentation, the students appeared to have some concerns about the activity. Therefore, their willingness to undertake the presentation and favor it was slightly less than four points. To explore how Taiwanese university students experienced intercultural presentation projects, their reflection responses were carefully and recursively examined. Recognitions, concerns, and impacts were identified as the main themes and their corresponding categories are presented in Table 2. Among different categories, “Increased motivation for preparation” and “Anxiety” stood out as expressed by the majority of the students. The analysis of the reflection responses is reported in the following sections.

**Table 2:** A coding scheme of student experience on videoconferencing presentations.

Theme	Categories	Frequency
Recognition	Meaningful language use	3
	Increased motivation for preparation	19
	Authentic nature of the interaction	4
Concern	Language issues	5
	Insufficient time for preparation	3
	Anxiety	14
Impact	Encouragement	4
	Cultural understanding development	6

#### 3.2.1. Recognitions

The students recognized different aspects of the cross-cultural presentation projects, namely opportunities to use English meaningfully, motivation to prepare for the project, and the authentic nature of the interaction.

##### 3.2.1.1. Meaningful language use

EFL learners who always interact with their non-native English-speaking classmates only in class rarely have the chance to interact with native speakers. The videoconferencing presentation was a great opportunity to practice English with more competent English speakers. As the participants mentioned,

“*I was excited about the project because I like the interaction with teachers in the United States, and it is a very unique and precious experience we could have*.” (Participant A).

“*We had foreign teachers from America. This made our presentation more meaningful. A good learning opportunity*.” (Student L).

##### 3.2.1.2. Increased motivation for preparation.

Since the participants viewed the presentation project to share Taiwanese cultures with the professors in the U.S. positively, they made considerable effort to prepare for it. They invested in different strategies to present their topics to audiences. First, the participants carefully planned their presentations and did their best to search online for relevant materials. They relied on their own experience and Internet searching to find relevant information. As the participant noted, “*[i]n order to prepare for the presentation, I did much online research and tried to recall my memories as much as possible*” (Participant J); “*I searched for the information and picked the most representative pictures for my topic*” (Participant H).

Next, some even relied on their personal connections to gather useful information for the presentation. Participant A mentioned “*[f]or the presentation activity, I collected all the information I needed online, asked for suggestions, and personally asked the bubble tea staff about its background story*.”

Moreover, the participants rehearsed their presentations several times and followed the steps of presentation preparation, which were taught at the beginning of the semester. They were motivated to impress their audience. These are the quotes from the participants: “*After making the PPT, I continued to confirm the script and practice every day*” (Participant E); “*I selected necessary information and wrote my presentation script. I did several rehearsals before the presentation. Meanwhile, I adjusted my PowerPoint slides and the script*” (Participant H).

##### 3.2.1.3. Authentic nature of the interaction.

The presentation project has provided EFL students with the experience to immerse themselves in engaging with unplanned discourse in the question-and-answer session at the end of the presentation. The findings showed that unplanned speech enabled the students to examine their communication efficacy and the audience’s understanding. It boosted their confidence to confirm that their hard work was rewarded due to their good performance in the question-and-answer session. For example, Participant A mentioned that “*the part I did very well in the presentation activity was that I answered the teacher’s questions very smoothly and fluently*.” Furthermore, Participant B said that “*I know I did a good job preparing for the presentation because I replied to their questions about stray animals fluently and calmly*.”

#### 3.2.2. Concerns

Three major concerns were identified: language issues, insufficient time, and affective filter.

##### 3.2.2.1. Language issues

The level of English proficiency was a main worry for the participants. Several of them pointed out that they were not confident enough in their command of grammar [“*The most serious issue is about grammar and vocabulary. Particularly, I had to improve my grammar”* (Participant B)] and pronunciation [*“I have to admit that I encountered struggles with the pronunciation”* (Participant O)].

Moreover, the question-and-answer session in the presentation made the participants anxious because it is unpredictable in nature. This made the participants worry about their ability to make instant responses in English. Participant J stated that “*[i]n the Q&A session, I found the teachers’ questions were the most difficult to understand, which made me feel stressed*.” Furthermore, Participant Q expressed that “the *teachers in America asked me many questions about the history and other questions about my topic. I really did not know how to answer in English*.”

##### 3.2.2.2. Insufficient time for preparation

To present their topic best, much time was invested in preparation [“*I needed to prepare a script and practice thousands of times in order to get fluent and flawless. It took a LOT of time*. (Participant K)”]. However, several participants mentioned that they had had little time to prepare for the presentation, although they really desired to perform well. They said, “*Little time to prepare the presentation. I had to think about it when I was working*” (Participant B) and “*I was willing to do my best for the presentation, but I have time issue to do so.”* (Participant Q).

##### 3.2.2.3. Anxiety

The next concern was about participant stage fright and language anxiety, which is viewed as “intense feelings of apprehension, tension, and even fear, when they think of foreign languages” (Ortega, 2009; p. 200). These negative emotions hampered the participants as numerous participants reported high tension during the presentations. Quotes are presented:

“*I am always nervous when I’m am on stage. My brain goes blank, and I feel it is difficult to speak anymore. I’m always afraid of questions that I can’t answer*.” (Participant F).

*“I was really nervous because it was my first time doing a lengthy presentation, particularly to foreign teachers.”* (Participant K).

*“I easily feel nervous when I need to speak in front of classmates and teacher so I usually perform poorly.”* (Participant L).

*“I knew that I could present well and I practiced this presentation, but I was too nervous and started stuttering.”* (Participant M).

*“I found it difficult to speak the sentence fluently. I think it’s because I get nervous when I go on stage, so I cannot speak properly.”* (Participant Q).

*“When I was speaking in English, I found that I would forget how to pronounce words because of nervousness.”* (Participant R).

*“I always feel afraid and nervous when I speak English.”* (Participant S).

#### 3.2.3. The impact of the presentation

The final theme was concerned with the impact that videoconferencing presentations had on the participants. Two categories fall under this theme: encouragement and cultural-understanding development.

##### 3.2.3.1. Encouragement

The participants expressed in the reflective journals that they made positive gains in the project. The sense of achievement and feedback they received from the project motivated them, and they were willing to invest more time out of their work time and put more effort into their journey in learning English. The participants stated,

“*From the teachers’ feedback, I realized that I must improve my grammar, and this cannot be changed quickly. So, I will continue studying hard*.” (Participant E).

“*I received much positive feedback from the teachers and peers. So encouraging! The American teachers said that I made a strong, lively, and informative speech. I will have another good presentation next time*!” (Participant K).

##### 3.2.3.2. Cultural understanding development

In the cross-cultural videoconferencing presentation, the interaction with authentic speakers enabled the participants to sharpen their target language, demonstrate their own cultures, and experience American culture. They noted that they learned more about their own cultures during the preparation of their presentations. They were eager to share their own cultures with the teachers in America and their local classmates. Participant L noted, “*I wanted to have a good presentation. My topic is not common in our culture. So I had to use a simple way to introduce but also need to make it detailed, complete and interesting, to make it understandable*.”

Furthermore, the participants felt proud and privileged to share their cultural backgrounds and cultural identity with the audience. For example, the participants mentioned that “*Their feedback was very important because they were literally viewing Taiwan’s culture from outsiders’ perspectives. So I wanted to promote our culture more* (Participant A),” and “*I introduced attractions about the transportation in Taiwan. I hope everyone, including the teachers in the United States, can understand Taiwanese culture and its beautiful landscape*” (Participant H).

## 4. Discussion and conclusion

This study aimed to explore how Taiwanese EFL university students viewed the cross-cultural videoconferencing presentation project. Novel to this study is to include presentations as main tasks and a cohort of professors in the United States as the presentation audience to interact with the students. This endeavor is crucial, given that this cross-cultural exchange brought authenticity to the EFL students’ language-learning task. The analysis of the participants’ attitude surveys and reflections enabled us to understand how they viewed the presentation task and interpreted their experience in the project.

The participants expressed positive viewpoints about the project, which corroborates evidence from previous observations (Jauregi and Bañados, 2008; Hsu and Beasley, 2019; Lenkaitis et al., 2019; Vurdien and Puranen, 2020). However, the participants’ favor and willingness to undertake the project were not as high as the perceived benefits the project brought to them. Possible explanations can be found in the participants’ reflections on the project.

The participants acknowledged several advantages brought by the project. Since videoconferencing creates a context similar to face-to-face interaction, users are able to communicate with each other spontaneously and interactively (Chun and Plass, 2000; Cirit-Işıklıgil et al., 2023). This is a significant key to the success of second-language learning (Gass and Selinker, 1994; Long, 2015). Furthermore, the participants appreciated that they felt encouraged to keep boosting their English-proficiency levels and gained more understanding of their own culture. Most importantly, all of the participants addressed in their reflection essays the increased motivation in preparing for the presentation, suggesting that the project involving target-language dominant faculty in the United States enabled the participants to highly engage in the learning task, which is in line with Hetrovicz (2021, 2022). These findings empirically highlight the need to include authenticity in the practice of language-learning tasks (Chamot, 2009; Long, 2015).

Yet, the participants’ concerns seemed to prevent them from concertedly participating in the project. Since they had to work in the daytime and had pre-intermediate proficiency levels, it was natural that some of them suffered from limited preparation time and had difficulties expressing their thoughts smoothly in English. The majority of participants particularly complained about their discomfort from language anxiety and stage fright during their presentations, which is consonant with Ibrahim and Hashim’s research (2021) that anxiety was frequently reported by second-language learners in videoconferencing projects. This may be explained by the nature of learning tasks in the current study. As oral presentations are an important part of the academic experience, the effectiveness of students’ presentations serves as a significant indicator of academic success (Prevoo et al., 2016). Second-language learners appeared to feel much more stressed when undertaking presentations (Hadi et al., 2021; Tsang, 2022). These negative emotions can impede learners’ second-language progress (Krashen, 1988) and should be addressed by educators and researchers to support students’ language learning.

The findings have implications for incorporating presentations through videoconferencing with cross-cultural exchanges in foreign-language teaching and learning. Firstly, the current study agrees with the argument made by researchers (Alshahrani, 2016; Sevy-Biloon and Chroman, 2019) that videoconferencing can be a useful venue for bringing authenticity to learners in advancing their language proficiency and cultural understanding. Through videoconferencing, interacting with audiences of different cultural backgrounds enables learners to explore their own cultures through the lens of others and practice their target language meaningfully and authentically. As supported by the culturally-relevant pedagogy, learners’ home and community cultural knowledge and practices are considered valuable resources in the classroom (Ladson-Billings, 1995; Morrison et al., 2008). Learners should be encouraged to maintain their own heritage while navigating the target language and its culture. Moreover, during the presentation, the spontaneity of real-time communication might raise anxiety. Instructors need to prepare students by teaching them coping strategies to handle such situations and minimize anxiety levels. Finally, based on student proficiency levels and schedules, instructional activities could be timely tailored to better fit student learning needs by providing students with different amounts of scaffolding throughout the project.

Nevertheless, the findings of the current study should be interpreted with caution. Its generalizability is limited due to its small sample size. The other limitation is that the participant made only one presentation. This single-activity factor could cause the “novel effect” to affect the participants’ attitudes. Future studies should include more participants and have a higher frequency of presentations. Furthermore, future studies need to explore the perspectives of teachers on such tasks, who play a crucial role in distance education (Ibáñez et al., 2022). More data collection approaches, such as interviews and classroom observations, can be implemented for the participants to elaborate on responses to the survey and reflection essays, thus further triangulating the research finding. Finally, the progress of participant presentation skills, English speaking ability, and inter-and intra-cultural awareness before and after a cross-cultural videoconferencing presentation project can be recorded.

## Data availability statement

The original contributions presented in the study are included in the article/supplementary material, further inquiries can be directed to the corresponding author.

## Ethics statement

Ethical review and approval was not required for the study on human participants in accordance with the local legislation and institutional requirements. The patients/participants provided their written informed consent to participate in this study.

## Author contributions

L-TY and H-PW conceptualization, formal analysis, methodology, project administration, and writing—review and editing. L-TY writing—original draft. All authors contributed to the article and approved the submitted version.

## Funding

The APC was funded by National Science and Technology Council, Taiwan, under grant numbers 111-2410-H-007-036.

## Conflict of interest

The authors declare that the research was conducted in the absence of any commercial or financial relationships that could be construed as a potential conflict of interest.

## Publisher’s note

All claims expressed in this article are solely those of the authors and do not necessarily represent those of their affiliated organizations, or those of the publisher, the editors and the reviewers. Any product that may be evaluated in this article, or claim that may be made by its manufacturer, is not guaranteed or endorsed by the publisher.
